# The Absence of Pyruvate Kinase Affects Glucose-Dependent Carbon Catabolite Repression in *Bacillus subtilis*

**DOI:** 10.3390/metabo9100216

**Published:** 2019-10-04

**Authors:** Joana Sousa, Philipp Westhoff, Karen Methling, Michael Lalk

**Affiliations:** 1 Institute of Biochemistry, University of Greifswald, 17487 Greifswald, Germany; joanasousa100@gmail.com (J.S.); pd.westhoff@gmail.com (P.W.); methling@uni-greifswald.de (K.M.); 2 Innovayt S/A, Av. João Paulo II 30, 4715-213 Braga, Portugal; 3 Institute of Plant Biochemistry, Heinrich-Heine-Universität Düsseldorf, Universitätsstraße 1, Düsseldorf, Germany

**Keywords:** *Bacillus subtilis*, metabolomics, pyruvate kinase, pyruvate, carbon catabolite repression, overflow metabolites

## Abstract

Pyruvate is a key intermediate of diverse metabolic pathways of central carbon metabolism. In addition to being the end product of glycolysis, pyruvate is an essential carbon distribution point to oxidative metabolism, amino acid and fatty acid syntheses, and overflow metabolite production. Hence, a tight regulation of pyruvate kinase (Pyk) activity is of great importance. This study aimed to analyze targeted metabolites from several pathways and possible changes in *Bacillus subtilis* lacking Pyk. Wild type and Δpyk cells were cultivated in chemically defined medium with glucose and pyruvate as carbon sources, and the extracted metabolites were analyzed by ^1^H-NMR, GC-MS, HPLC-MS, and LC-MS/MS. The results showed that the perturbation created in the pyruvate node drove an adaptation to new conditions by altering the nutritional compounds’ consumption. In Δpyk, pyruvate, which is subject to glucose-dependent carbon catabolite repression, did not comply with the hierarchy in carbon source utilization. Other metabolic alterations were observed such as the higher secretion of the overflow metabolites acetoin and 2,3-butanediol by Δpyk. Our results help to elucidate the regulatory transport of glucose and pyruvate in *B. subtilis* and possible metabolic reroute to alternative pathways in the absence of Pyk.

## 1. Introduction

The bacterium *Bacillus subtilis* is ubiquitous in nature, living primarily in the soil and associated with water sources, and can successfully adapt to changes in the environment [1,2]. Being the best characterized member of Gram-positive bacteria, *B. subtilis* is currently the focus of interest in biotechnology and industry due to its easy genetic manipulation, the availability of extensive physiological and biochemical data, and due to being an efficient producer of economically valuable metabolites [1,3,4]. In this context, knowledge of metabolomic flux pathways and, consequently, improved strategies for the overproduction of desired metabolites have made *B. subtilis* a potent applicant organism in a wide industrial field. Pyruvate is a key intermediate of various central carbon metabolism pathways, acting as a branching point of glycolysis and tricarboxylic cycle (TCA cycle), and a substrate of fatty acid and amino acid synthesis. The metabolic link between the glycolysis and the TCA cycle is represented by the phosphoenolpyruvate (PEP)–pyruvate–oxaloacetate node, which directs carbon flux and acts as a switch point for carbon flux distribution within the central metabolism [5]. Furthermore, poor coordination between glucose consumption and precursor synthesis in the TCA cycle has been seen as one of the responsible causes for the incomplete oxidation of glucose and production of alternative metabolites—namely, overflow metabolites [6,7,8]. Therefore, the control of pyruvate homeostasis and its fate is of great importance for cell robustness and viability during environmental changes.

It is well known that in *B. subtilis*, preferred carbon sources, such as glucose, are able to repress the transport and catabolism of alternative substrates. This carbon catabolite repression (CCR) is achieved through the transcriptional control of genes required for the utilization of secondary carbon sources, imposing a hierarchy in the use of the available nutrients and preventing the waste of resources. Different mechanisms exist in bacteria for the signal transduction pathways that lead to the CCR. In *B. subtilis*, the CCR is mediated by the phosphotransferase system (PTS). PTS is a multiprotein phosphorelay mechanism that catalyzes the phosphorylation of incoming sugar substrates and their simultaneous translocation across the cell membrane. The CCR is also mediated by the catabolite control protein A (CcpA), a pleiotropic transcription factor and a master regulatory protein, which can also function as a carbon catabolite activator (CCA) in certain genes, the histidine-containing protein HPr of the PTS, and the bifunctional HPr kinase/phosphorylase (HPrK) [9,10,11,12,13].

During sugar uptake, a chain reaction is triggered inside the cells, where, after successive phosphorylation, a phosphoryl group is transferred to the His15 residue of the HPr. Ultimately, the HPr-His-P transfers the phosphoryl group to the enzymatic transporter EII, allowing the uptake of glucose. Furthermore, it is known that when the intracellular concentration of fructose bisphosphate (FbP) is high, HPr can also be phosphorylated at Ser46 by the HPr kinase/phosphorylase (HPrK). This reaction allows the binding of HPr-Ser-P to CcpA. The HPr–Ser–P–CcpA interaction permits the binding of CcpA to specific sites on the DNA and, thereby, represses or activates the transcription of determined genes [10,11].

Although extensive studies have been carried out concerning the CCR phenomenon, the characterization of *B. subtilis* cultivated in pyruvate and carbon source co-utilization studies using pyruvate are still scarce.

Recently, a pyruvate transport system was identified and characterized in *B. subtilis* [8,14,15]. Charbonnier and co-workers verified that the preferred carbon sources in *B. subtilis*, glucose or malate, trigger the binding of CcpA upstream of the *pftAB* gene, which encodes a pyruvate facilitated transporter, resulting in the repression of pyruvate utilization. When these preferred carbon sources are absent, CcpA repression is diminished [8]. Furthermore, a two-component regulatory system named LytST is also involved in pyruvate metabolism and uptake through the membrane. LytST induces the expression of *pftAB* in the presence of external pyruvate. However, when the pyruvate flux into the cells is high, *pftAB* transcription is retro-inhibited via LytST. It was also shown that LytST activity works in a pyruvate dose manner, which permits the balance of intracellular pyruvate levels and adaptation to environmental changes [8]. The ability to use diverse carbon sources represents a key adaptation mechanism that allows cells to thrive in their natural habitat. Building on this knowledge, we became interested in how pyruvate is metabolized. Specifically, these observations raise the question of whether a co-assimilation of pyruvate and the preferred carbon source, glucose, from the medium would change in the presence of a high pyruvate gradient. So far, little is known concerning the absence of Pyk, how this affects the uptake and secretion patterns of pyruvate and other carbon sources in *B. subtilis*. Thus, we wanted to provide deeper insights into the physiology and metabolic responses of *B. subtilis* to the absence of pyruvate kinase (Pyk), and, ultimately, give hints towards the possible rerouting of carbon fluxes through alternative pathways. To do so, a combination of ^1^H-NMR spectroscopy as well as chromatographic separation coupled with mass spectrometry (GC-MS, HPLC-MS, and LC-MS/MS) was applied, allowing the identification of the extra and intracellular metabolites of wild type (wt) and Δpyk cells.

## 2. Results

### 2.1. Concomitant Consumption of Glucose and Pyruvate by ΔPyk—Carbon Catabolite Repression of Pyruvate Uptake in the Presence of Glucose Was Relieved

**Figure 1:**
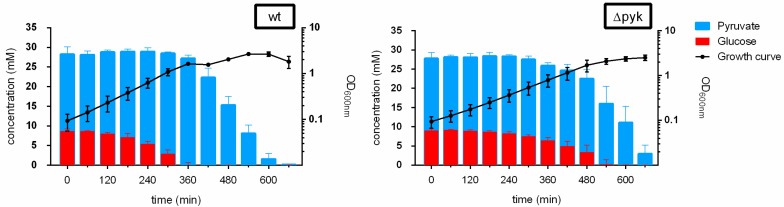
Growth curves and extracellular concentrations of glucose and pyruvate of *Bacillus subtilis* wild type (wt) and pyruvate kinase (Δpyk) under M9GlcPyr medium cultivation. Black lines illustrate the growth curves, while colored columns represent the external concentration of glucose (red) and pyruvate (blue) at each hour. Data are represented as the mean values ± standard deviation (SD) of 4 biological replicates. M9GlcPyr—a mixture of glucose and pyruvate.

For the analysis of *B. subtilis* wt and Δpyk, cells were cultivated in a chemically defined medium with a mixture of glucose and pyruvate (M9GlcPyr) as carbon sources. The growth curves arising from this condition are represented in Figure 1 (black lines).

Pyk mutant cells showed a growth delay during the exponential phase when compared to wt, even though both strains reached the same maximum optical density (OD) (OD 2.5) (Table S1). For wt, a diauxic growth curve was visible with an intermediate stationary phase between 360 and 420 min at OD 1.6.

**Figure 2:**
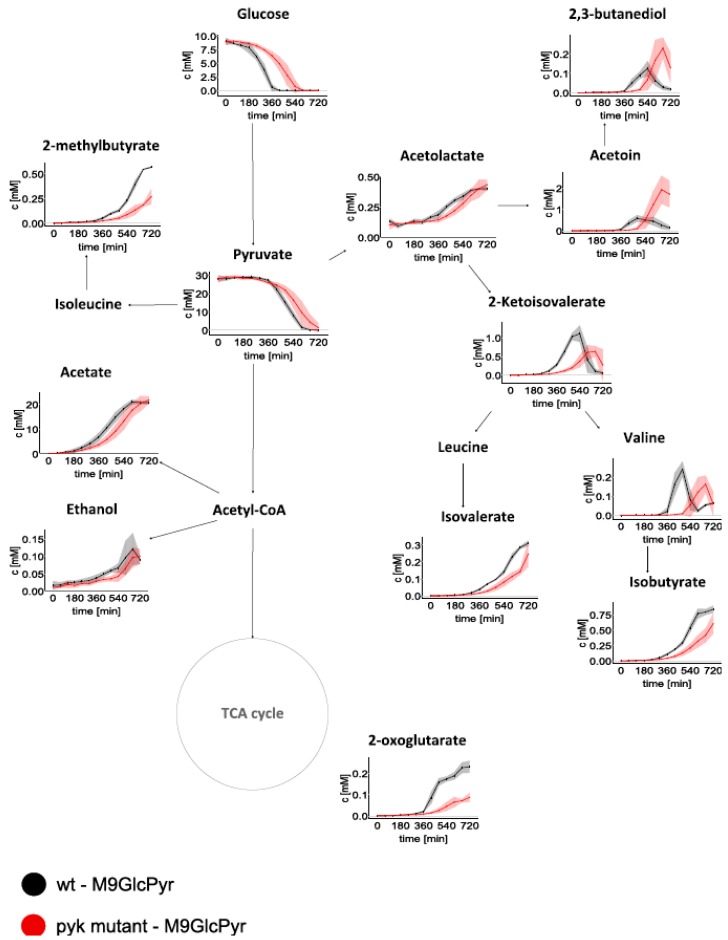
Time-resolved extracellular metabolites under M9GlcPyr medium cultivation. Absolute concentrations of consumed and secreted metabolites by wt (black) and Δpyk (red) are displayed. Data are represented as the mean concentrations ± SD (shaded) of quadruplicate samples.

Using Nuclear Magnetic Resonance (NMR) spectroscopy technique, changes in the concentration of extracellular metabolites were monitored in a time-dependent manner (Figure 2).

These results revealed that, in the wt, pyruvate consumption was initiated at 360 min of cultivation, when glucose was depleted from the medium (Figure 1 and Figure 2, and Table 1). On the contrary, pyruvate uptake in Δpyk started at 360 min, when 6.4 ± 0.8 mM of glucose was still available, showing a concomitant consumption of both carbon sources.

Interestingly, acetate was accumulated in similar concentrations in both strains and no uptake was detected until the end of cultivation (Figure 2).

Other typical overflow metabolites produced by *B. subtilis*, such as acetoin and 2,3-butanediol, were detected in both cultivations. However, these were secreted in notably higher amounts in Δpyk, than in the wt (Table S2). In both strains, these metabolites were taken up when external glucose was exhausted. The wt secreted significantly larger amounts of 2-oxoglutarate, valine, and branched-chain amino acid (BCAA) degradation products such as 2-ketoisovalerate, isobutyrate, 2-methylbutyrate, and isovalerate. Moreover, the concentration of valine and 2-ketoisovalerate started to drop when glucose was completely taken up in both strains.

### 2.2. Intracellular Metabolome Revealed Several Central Metabolic Pathways Altered in ΔPyk

For the investigation of possible metabolic changes in *B. subtilis* lacking Pyk, the intracellular metabolome was also monitored under the same growing conditions. During the exponential growing phase, wt and Δpyk cells were harvested and quadruplicate samples were inspected using GC-MS, LC-MS, and LC-MS/MS.

**Figure 3:**
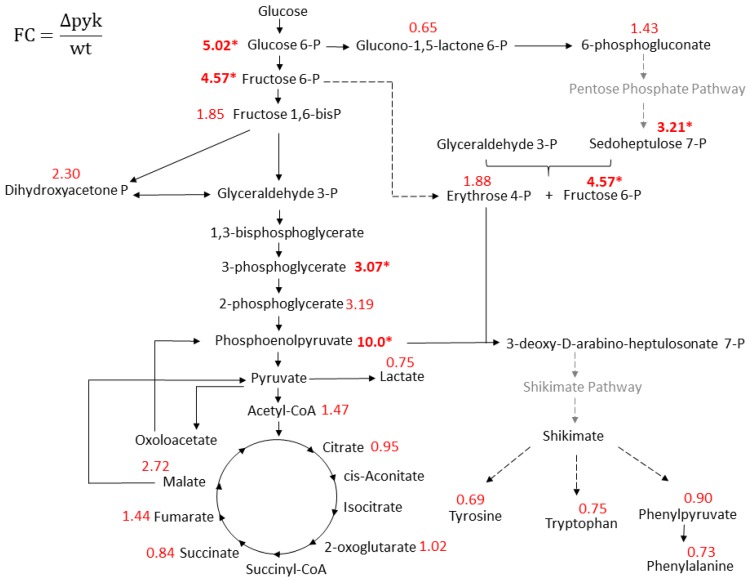
Fold change (FC) of some intracellular metabolites from central metabolism. Relative levels of metabolites from central metabolism for Δpyk as compared with wt under M9GlcPyr cultivation. FC was determined using the relative quantification of four biological replicates. Significant alterations (*p*-value ≤ 0.05) are marked in bold and asterisk.

Altogether, 92 metabolites from several metabolic pathways were identified and relatively quantified (Table S3). Several metabolites showed altered concentration levels when both strains were compared. The major differences were the notably increased levels of glycolytic metabolites in Δpyk, such as phosphoenolpyruvate (PEP), with a fold change (FC) of 10, followed by glucose 6-phosphate (FC 5.0), fructose 6-phosphate (FC 4.6), 3-phosphoglycerate (FC 3.1), and 2-phosphoglycerate (FC 3.2) (Figure 3).

Considering the Pentose Phosphate Pathway (PPP), sedoheptulose 7-phosphate presented significant (*p*-value ≤ 0.05) altered concentration levels (FC 3.2). In the TCA cycle, although 2-oxoglutarate secretion during late exponential and stationary phase was lower in Δpyk, the intracellular concentration at 0.5 OD—when intracellular sampling was conducted—was similar in both strains (FC 1.0).

**Figure 4:**
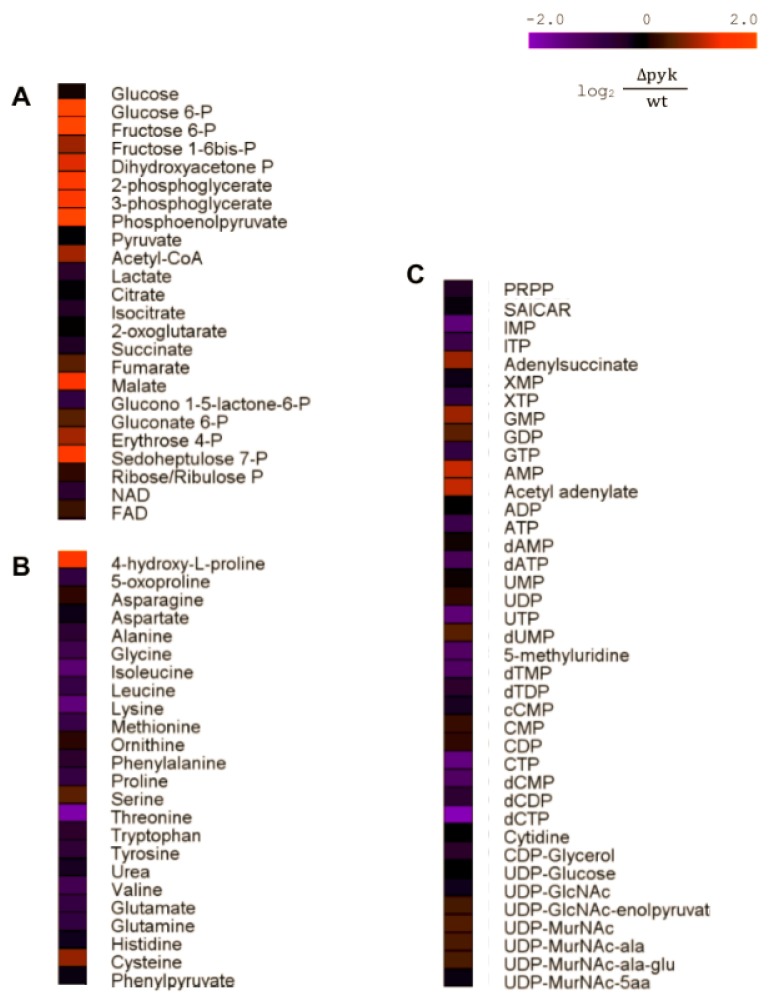
Heat map of intracellular metabolite levels of glycolysis, tricarboxylic (TCA) cycle and Pentose Phosphate Pathway (PPP) (**A**), amino acids (**B**), and intermediates of purine and pyrimidine metabolism and cell wall precursors (**C**), in wt and Δpyk under M9GlcPyr cultivation. The color code represents the log_2_ FC between Δpyk and wt, whereas increased levels are indicated in orange and lower levels in purple. Data are shown as the mean of four biological replicates. P: phosphate.

Among the nucleotides, nucleoside triphosphate uridine triphosphate (UTP) and cytidine triphosphate (CTP) were determined in significantly lower amounts in Δpyk (FC 0.40 and 0.38, respectively), as well as inosine monophosphate (IMP) (FC 0.40) and the deoxy nucleotides deoxycytidine triphosphate (dCTP (FC 0.27) and deoxythymidine monophosphate (dTMP) (FC 0.47). On the contrary, adenosine-monophosphate (AMP) was the only nucleotide with statistically higher levels in Δpyk (FC 2.1) (Figure 4 and Table S3).

## 3. Discussion

### 3.1. Carbon Catabolite Repression of Pyruvate Uptake in ΔPyk Cells Was Relieved

The present study comprehensively monitored changes in the intra and extracellular metabolites of *B. subtilis* but also the alterations in the metabolite uptake and secretion triggered by the absence of Pyk. An interesting outcome of this study is that pyruvate utilization was subject to glucose-dependent CCR in wt cells, while this repression was not observed in Δpyk. The rise in pyruvate influx in wt cells initiated at 360 min of growth, only after external glucose was depleted, indicating that pyruvate was susceptible to glucose-dependent CCR. This phenomenon is also perceived by the diauxic growth curve obtained, not described so far. On the other hand, in Δpyk, 6.4 ± 0.8 mM of glucose was still available in the medium when pyruvate consumption was rapidly initiated. The CCR derepression seen in Δpyk can be hypothesized as a cellular metabolic response for the immediate need of other carbon sources, since the central metabolic pathway is possibly altered due to the accumulation of glycolytic metabolites (discussed below).

A pyruvate transport mechanism regulated by the master regulator CcpA was recently discovered. The CcpA controls the expression of *pftAB* that encodes a pyruvate facilitated transporter in accordance with the absence/presence of glucose or malate. With the knockout and overexpression of *pftAB*, it was concluded that the pyruvate gradient observed between the outside and the inside of the cell drives the facilitated PftAB-mediated transport of pyruvate across the cell membrane [8]. In our study, when Δpyk cells are growing in M9GlcPyr and the intracellular pyruvate pool is presumably low, LytST might respond by inducing *pftAB* transcription sense due to the high pyruvate gradient concentration, leading to a rapid increase in pyruvate influx. In wt, where the pyruvate concentration is under physiological levels inside the cells and the pyruvate gradient is lower, LytST induction is probably impaired and/or the repression of *pftAB* by CcpA is highly active. When glucose is completely consumed, the CCR effect on *pftAB* is relieved and wt cells are able to take up pyruvate as a nutrient source.

Preliminary studies were also conducted under cultivation with glucose as the single carbon source (data not shown). In these, the deletion of *pyk* resulted in the impairment of cell growth, a slow consumption of glucose from medium, and an accumulation of intracellular FbP (FC 5.3), when compared to the wt. It is speculated that a possible cause for the low glucose consumption rate seen in Δpyk is the impairment of glucose uptake by the PTS system due to the accumulation of high levels of FbP. The concentration of FbP has been proposed to regulate the PTS system at the level of EII-mediated uptake. A high concentration of FbP directly stimulates the phosphorylation of HPr through HPrK, favoring the formation of the HPr–Ser–P–CcpA complex [16,17]. Further experiments are necessary to clarify the glucose dynamics in Δpyk and the possibility of the PEP-dependent phosphotransferase system being impaired. These should include the inspection of other PTS sugar transport by Δpyk, the quantization of HPr-Ser-P, and the HPrK activity levels.

### 3.2. The TCA cycle and the Pentose Phosphate Pathway (PPP) Are Possibly Altered in Δpyk

In the initial exponential growth of wt cells with available glucose and pyruvate, no TCA cycle metabolites were detected outside the cells, which is predicted to be weakly active during this growth phase [18]. In *B. subtilis*, when preferred carbon sources are available in the medium, the activity of the first TCA cycle enzymes is inhibited by several regulatory proteins [10,19]. When the external glucose was depleted, an efflux of 2-oxoglutarate in wt was initiated (Figure 2), suggesting an increase in TCA cycle activity in response to cell requirements. At this time point (360 min), pyruvate consumption also begun, which can be used for replenishment of the TCA cycle.

As previously discussed, during the late exponential growth phase of Δpyk, pyruvate uptake is initiated when glucose is still available. Notably, at this time, 2-oxoglutarate secretion begins, reaching 30% of the concentration seen in wt (0.23 ± 0.03 mM and 0.07 ± 0.01mM for wt and Δpyk, respectively) at the end of cultivation. Nevertheless, in stationary phase, TCA cycle repression was possibly relieved for the use of alternative carbon sources in the central metabolic pathways. Thus, pyruvate that is crossing into the cell is oxidized to acetyl-CoA production or catabolized by pyruvate carboxylase to oxaloacetate, and immediately directed to the TCA cycle.

As previously described, the growth of Δpyk resulted in accumulation of glucose 6-phosphate. It has been reported that the lack of Pyk in *Escherichia coli* and *B. subtilis* under glucose medium increases the flux of glucose 6-phosphate through the PPP [20,21,22,23]. Although just a few metabolites of this pathway were identified by our method, the results are consistent with this finding, since sedoheptulose 7-phosphate showed higher amounts in Δpyk. The perturbation seen in glycolysis in the mutant could have caused the reroute of flux metabolism to compensate the required amount of NAD(P)H and synthesis of nucleotide precursors [24,25].

### 3.3. Overflow Metabolites: Increased Secretion of Acetoin and 2,3-Butanediol by Δpyk

Common phenomena during *B. subtilis* cultivation with high availability of carbon sources are the production and secretion of overflow metabolites, even in aerobic conditions. Studies suggest that the onset of overflow metabolism during a surplus of carbon and energy is driven by the saturation of the electron transport chain that generates the demanding ATP in fast-growing cells, and the imbalance of the intracellular NADH/NAD^+^ ratio observed prior to the beginning of fermentation [26,27].

As typical in *B. subtilis*, acetate was the overflow compound with the highest secretion amount [24]. The expression of the genes involved in the synthesis of acetate is activated by CcpA in the presence of glucose (CCA effect). Moreover, the gene involved in the utilization of acetate is subject to CCR [10,28,29]. Consistently, acetate was secreted as soon as glucose was imported and metabolized by both strains. When the stationary phase was reached, acetate uptake was not noticed, as commonly reported [11,30]. Although acetate could be reimported and utilized as an alternative carbon source after the depletion of glucose (and the consequent diminish of the CCR effect), cells preferred to utilize pyruvate. Thus, acetate efflux continued until the end of cultivation. Yet, it is speculated that acetate influx could be observed if cells were cultivated for longer. Furthermore, it is also hypothesized, that acetate uptake could have been subject of catabolic repression by pyruvate since the latter was consumed as an alternative carbon source during the exponential phase.

Acetoin and 2,3-butanediol secretions were also detected. Notably, the effluxes were much higher in Δpyk than in wt. The reason for these differences remains unclear. The upper high efflux of acetoin was previously observed [31]. The production of these overflow metabolites is perceived as a preventive mechanism of environment acidification due to acetate accumulation [29,32]. Acetoin is synthesized from pyruvate and can either be secreted or converted into 2,3-butanediol in order to generate NAD^+^. These compounds can also be reimported during the stationary phase and, in this way, are used as an energy-storing strategy. The fact that Δpyk secreted more acetoin and 2,3-butanediol than in wt leads us to think that the perturbation occurring in the pyruvate node could result in the imbalance of the NADH/NAD^+^ ratio, which is known to be potentially toxic to the cells [26,33]. Consistently, the higher synthesis of acetoin and subsequent 2,3-butanediol results in more NAD^+^ molecules being regenerated, which can help balance the reducing power ratio. Likewise, the secretion of acetoin and, to an extent, 2,3-butanediol occurred alongside acetate accumulation, which is in agreement with the fact that acetoin synthesis can also be induced by acetate [32,34,35]. On the contrary to acetate, acetoin and 2,3-butanediol uptake was observed when the stationary phase was reached, suggesting that they were used as alternative carbon sources as soon as glucose and pyruvate were exhausted.

For a robust statistical analysis, these studies were performed using four biological replicates, instead of the common use of triplicates. Nevertheless, statistical corrections could have been applied, such as Bonferroni or Sidak methods, to increase the power of the statistical outcomes.

These findings may shed light on the mechanisms of *B. subtilis* to regulate the transport of glucose and pyruvate and the possible metabolic reroute to alternative pathways in the absence of Pyk.

## 4. Materials and Methods

### 4.1. Bacterial Strains and Growth Conditions

Cloning and genetic manipulations were conducted using standard procedures [36,37]. *B. subtilis* strains were derived from BSB1, a tryptophane (trp^+^) derivative of *B. subtilis* 168. The deletion of the *pyk* gene was performed in the Department of General Microbiology, Georg-August-Universität Göttingen with the long flanking homology PCR technique, as previously described by Pietack et al. [38,39]. This was achieved by transformation with PCR products constructed using oligonucleotides to amplify DNA fragments flanking the target genes and an intervening antibiotic resistance cassette. For that purpose, genes that mediate resistance against erythromycin were amplified from the plasmid pDG646 [40]. The PCR product was purified using the QIAquick PCR Purification Kit (Qiagen; Hilden; Germany). *B. subtilis* was transformed with the purified PCR products and transformants were selected on plates with the antibiotic. Clones were examined by checking PCR for the integrity of the resistance cassette. The DNA sequence of the flanking regions was verified by sequencing. Both *B. subtilis* BSB1 wild-type (wt) and pyruvate kinase mutant (Δpyk) were grown in Lysogeny Broth (LB) and M9 media was prepared as previously described by Harward and Cutting with small changes [36]. To prevent precipitation problems, magnesium sulfate (1 M) and calcium chloride (100 mM) were added as separated solutions. Moreover, iron chloride solution (50 mM) was prepared with citric acid (100 mM) and was also added as a separate solution. For growth experiments, different carbon sources were added to the M9 stock medium: (i) glucose (20 mM) and malate (5 mM) (M9GlcMal); (ii) glucose (10 mM) and pyruvate (60 mM) (M9GlcPyr).

### 4.2. Cultivation

LB agar plates of *B. subtilis* were prepared from frozen stocks (−80 °C, in 15% (*v*/*v*) glycerol) and incubated overnight at 37 °C. For genetic selection, kanamycin (3 µg/mL) was added to the mutant plates.

The pre-cultures were incubated from isolated colonies for 4 h in 5 mL of LB medium at 37 °C and 300 rpm. These cultivations were used to prepare a second set of overnight cultures in M9GlcMal in several dilutions to ensure it was in the exponential growth phase. After 15 h of incubation at 37 °C and 260 rpm, the cultures in exponential growth—optical density (OD_600nm_) between 0.5–0.8—were centrifuged at 6000 rpm and 4 °C for 3 min (Heraeus Multifuge X1R, Thermo Scientific, Waltham, MA, USA). The cells were resuspended in the main culture medium and were inoculated with an initial OD_600nm_ of 0.1 at 37 °C and 300 rpm.

### 4.3. Sampling of Extracellular and Intracellular Metabolites

To investigate the extracellular metabolite composition, 2 mL of bacterial culture was sterile filtered (0.45 µm pore size, Filtropur S, Sarstedt AG) every 60 min and stored at −20 °C prior to ^1^H-NMR measurements.

For intracellular metabolite samples, 20 OD units of cell culture were harvested via a vacuum-dependent fast-filtration system as described by Meyer et al. with few modifications (Figure S1) [41].

In brief, the main culture was transferred into a falcon tube and cooled by dipping the sample periodically in liquid nitrogen for 10 s maximum (approximately 1 s each time). During this in/out of liquid nitrogen cycle, the sample was carefully shaken to avoid freezing and metabolite leakage caused by cell lysis. Subsequently, the cooled cell culture was filtered (regenerated cellulose membrane filter, 0.45 µm pore size, 100 mm diameter, RC55 Whatman) and washed 2 times with isotonic sodium chloride solution at 4 °C (0.8 or 0.9% when cultivated in chemically defined or complex medium, respectively). The filter was immediately transferred to a falcon tube containing 5 mL of ice-cold extraction solution (60% *w*/*v* of ethanol absolute 99.8%) and the internal standard (ISTD) constituted of 2.5 nmol of camphor sulfonic acid (CSA) for HPLC-MS, 20 nmol of labeled amino acids (Cell Free Amino Acid Mixture—^13^C, ^15^N; Sigma Aldrich, St. Louis, MO, USA) for LC-MS/MS, and 20 nmol of ribitol for GC-MS analysis. The metabolites were quenched by freezing the sample immediately in liquid nitrogen. The falcon tube was stored at −80 °C until extraction. Subsequently, the sample was snap-frozen in liquid nitrogen. The sampling procedure took less than 1 min. The efficient quenching of the metabolism was confirmed with the determination of the adenylate energy charge (AEC) of each biological sample, according to the method of Atkinson [42]. In both strains, the AEC was over 0.7 (data not shown).

The sample was stored at −80 °C until extraction. For cell disruption and metabolites extraction, 10 freeze/thaw cycles were performed by alternately thawing it on ice, vortexing and shaking the samples. Afterwards, the sample was centrifuged for 5 min at 4 °C and 13,000 rpm. The supernatant was collected in a new falcon tube and left on ice. The pellet formed was extracted once again with 5 mL of water (HPLC-MS grade). The two supernatants were combined and restocked with distilled water to a final organic solution concentration of 10% and stored at −80 °C prior to lyophilization. The sample was lyophilized with a Christ Alpha 1–4 LSC lyophilizer at −52 °C and 0.25 mbar. To avoid disturbances in HPLC columns associated with the accumulation of macromolecules (i.e., proteins), a third extraction was added to the experimental procedure. Therefore, 500 µL of cold water (HPLC-MS grade) and 100 µL of cold chloroform (HPLC-MS grade) were added to the sample, followed by 1 min of periodically shaking and vortexing. Afterwards, sample was stored at −20 °C for 10 min. The supernatant was separated by centrifugation for 10 min at 4 °C and 13,000 rpm. The aqueous phase was collected and lyophilized once again. The sample was stored at −20 °C until analytical analyses.

### 4.4. H-NMR Spectroscopy Measurement and Data Analysis of Extracellular Metabolites

A volume of 400 µL of sample was mixed with 200 µL of sodium hydrogen phosphate buffer (0.2 mM, pH 7.0 made up with 50% D_2_O) containing 3-trimethylsilyl-[2,2,3,3-D_4_]-1-propionic acid (1 mM) to provide a nuclear magnetic resonance NMR-lock signal. All NMR spectra were obtained at 600.27 MHz at 310 K using a Bruker Avance-II 600 NMR spectrometer operated by TOPSPIN 3.2 software (Bruker Biospin GmbH, Rheinstetten, Germany). A modified 1D-^1^H-nuclear Overhauser effect spectroscopy (1D-NOESY) pulse sequence was used with presaturation on the residual peak (HDO) during both relaxation delay and mixing time. A total of 64 free induction decays (FID scans) were collected, using a spectral width of 30 ppm for a one-dimensional spectrum.

The identification and quantification analysis were carried out using AMIX v3.9.11 software (Bruker Biospin GmbH, Rheinstetten, Germany). The signal peak identification was based on spectra alignment of pure standard compounds (Sigma-Aldrich, St. Louis, MO, USA). Quantification was carried out by integration and comparison of designated peaks to an external standardized computed 10 mM signal at 15 ppm. Unidentified signals were relatively quantified due to their unknown quantity of protons.

### 4.5. GC-MS Measurement and Data Analysis of Intracellular Metabolites

The dried samples were derivatized firstly with 60 µL of methoxyamine (20 mg/mL pyridine) for 90 min at 37 °C and secondly with 120 µL of *N*-methyl-*N*-trimethylsilyltrifluroacetamide (Chromatographie-Service GmbH) for 30 min at 37 °C. Samples were centrifuged for 2 min at room temperature and the supernatant was transferred into GC-vial for injection. GC-MS analysis was performed with an Agilent 6890N GC system with an auto-sampler G2614A model coupled to a mass selective detector 5973N model (Agilent Technologies, St. Clara, CA, USA). A 2 µL sample was injected (G2613A model Series Injector) with a split 1:10 at 250 °C, using helium as the carrier gas (split flow of 10 mL min^−1^ and 8.8 Psi).

The chromatographic run was performed as described by Dörries et al. [43]. Using a 30 m DB 5-column (JW Scientific, Folsom, CA, USA) with 0.25 mm inner diameter and 2.5 µm film thickness, and a constant gas flow of 1 mL/min HPr–Ser–P–CcpA complex. The oven program started with an initial temperature hold at 70 °C for 1 min and continued with a heating rate of 1 °C min^−1^ up to 76 °C, 5 °C min^−1^ up to 220 °C, and 20 °C min^−1^ up to 330 °C, with a hold for 3 min followed by a 10 min isothermal cool-down to 70 °C. The analytes were transferred to a quadrupole mass analyzer operated in the electron impact ionization (EI) mode with an ionization energy of 70 eV. Data acquisition was carried out in 40 min runtime. Full scan mass spectra were acquired from 50 to 500 *m*/*z* at a rate of 2 scans/sec and with a 6 min solvent delay.

The qualitative analysis of the detected compounds was performed using ChromaTOF software (LECO Corporation, St. Joseph, MI, USA). Metabolite identification was carried out by comparison of retention time and fragmentation patterns peaks detected to the NIST mass spectral database 2.0 (Gaithersburg, MD, USA) and an in-house database. For relative quantification, the area of the quantifier ion of each metabolite was integrated and normalized to the area of the ISTD (ribitol).

For precision analysis control, daily quality control (dQC) samples were analyzed during the batch. The dQC consisted of 53 metabolites with 100 nmol concentration for each metabolite. Precision analysis was determined by assessing the measured dQC in calibration curves with concentrations ranging from 0.5 nmol to 500 nmol of each metabolite. The calibration curves were fit with a polynomial of degree 2 and 1/x weighting based on minimum of 6 calibration points.

### 4.6. HPLC-MS Measurement and Data Analysis of Intracellular Metabolites

For the intracellular metabolite analysis in HPLC-MS, the lyophilized samples were dissolved in 100 µL of water (HPLC-MS grade) and centrifuged for 2 min at room temperature. The supernatant was transferred into a HPLC vial for injection. The HPLC-MS analysis was carried out using an Agilent 1100 HPLC system consisted of a degasser, a quaternary pump and a G1329A autosampler with controlled temperature coupled to Bruker micro-TOF mass spectrometer (Bruker Daltonics, Bremen, Germany).

Chromatography was performed on a SymmetryShield RP18 column (3.5 µm, 150 × 4.6 mm) (Waters, Milford, MA, USA) with a SecurityGuard cartridge C18 pre-column (4 × 3.0 mm) (Phenomenex, Torrance, CA, USA) using an ion-pairing reagent and a methanol gradient as described by Dörries et al. [43]. In detail, the mobile phase consisted of eluent A: 95% water and 5% methanol, containing 10 mM of tributylamine as the ion-pairing reagent and 15 mM of acetic acid, pH 4.9; and eluent B: 100% methanol. Data acquisition was carried out in 42 min runtime with a flow rate of 0.4 mL min^−1^. The gradient elution started with 100% A for 2 min, 0–31% B in 2 min and continued with 31 to 50% in 18 min. Followed by 50–60% B in 2 min, 60–100% B in 1 min and left 100% for 7 min. The eluent A returned to 100% in 1 min and was left for 10 min until the end of the run. The gradient elution started with 100% A for 2 min, 0–31% B in 2 min and continued with 31 to 50% in 18 min. Followed by 50–60% B in 2 min, 60–100% B in 1 min and left 100% for 7 min. The eluent A returned to 100% in 1 min and was left for 10 min until the end of the run.

Mass spectrometry was operated in electrospray ionization and negative-ion mode using a mass scan range of 50 to 3000 *m*/*z*. Internal MS calibration was carry out in the beginning of each chromatographic run with 16 different masses from a sodium formate solution tune mix (49.4% water, 49.4% isopropanol, 0.2% formic acid, and 10 mM sodium hydroxide).

Metabolite identification was carried out by comparison of retention time and *m*/*z* values of detected peaks ([M − H]^−^ or [M − 2H]^2−^) with database alignment of the calculated exact mass.

The quantitative analysis was carried out using QuantAnalysis (Bruker Daltonik, Bremen, Germany). The extracted ion peaks were integrated and normalized to the ISTD (CSA) area.

The dQC samples consisted of 22 metabolites with 10 nmol concentration of each metabolite. Precision analysis was determined by assessing the measured dQC in calibration curves with concentrations ranging from 0.5 nmol to 500 nmol of each metabolite. The calibration curves fitting was performed with a polynomial of degree 2 and 1/x weighting based on a minimum of 6 calibration points.

### 4.7. LC-MS/MS Measurement and Data Analysis of Intracellular Metabolites

The amino acids proline, arginine, and citrulline were analyzed without derivatization on an Intrada amino acid column (50 × 3 mm, 3 µm) (Imtakt Corporation) with acetonitrile/100 mM ammonium-formate (20/80, *v*/*v*) as eluent A and acetonitrile/THF/25 mM ammonium formate/formic acid (9/75/16/0.3, *v*/*v*/*v*/*v*) as eluent B.

A 2 µL sample was injected in the column stabilized at 40 °C. Data acquisition was carried out in 26 min runtime with a flow rate of 0.4 mL min^−1^.

The gradient elution started with 100% B for 5 min, followed by 0–17% A in 4.45 min. Eluent A reached 100% in 1.15 min and was left in this condition for 6 min. Afterwards, eluent B returned to 100% in 50 sec and was left for 8 min until the end of the run.

Analyses were performed on a HPLC system (1200) consisting of a degasser, a binary pump, a temperature-controlled autosampler, and a column oven coupled to a 6460 triple quadrupole mass spectrometer with Electrospray ionization (ESI) Jet stream source (all Agilent Technologies). The mass spectrometer was operated in the positive mode (Table S4) using multiple reaction monitoring (MRM).

Proline—^13^C, ^15^N and arginine—^13^C, ^15^N were used as internal standard compounds for relative quantification (Cell Free Amino Acid Mixture—^13^C, ^15^N; Sigma Aldrich).

Quantification was carried out using Mass Hunter Quantitative Analysis (for QQQ) B 06.00 from Agilent.

### 4.8. Statistical Analysis and Visualization

Microsoft Excel software 2007 was used for metabolite quantification and calculation of standard deviations (SD) and fold changes (FCs).

The visualization of the time-resolved extracellular metabolite changes was performed using Excel and VANTED software v2.01.

The heat map of metabolites was generated using MeV v4.8.1 with hierarchical clustering analysis using Euclidean distance and average linkage method.

Bar charts, XY plots were created using GraphPad PRISM software v6.01. Unpaired t-tests were also carried out in GraphPad PRISM. The two-sided homoscedastic *t*-tests were used to calculate *p*-values, whereas *p*-values ≤ 0.05 were considered as being statistically significant.

The area of *m*/*z* detected for each metabolite in the spectrometric techniques was integrated and normalized to the integral of the area of *m*/*z* the internal standard by using, resulting in the relative metabolite amount per 20 OD units.

The metabolite missing values were replaced with half the minimum positive value in the original data.

## 5. Conclusions

This study shows the metabolic responses of *B. subtilis* lacking Pyk. We were able to highlight relevant metabolic differences between wt and Δpyk cells when monitored in a chemically defined medium with glucose and pyruvate as carbon sources.

In this work, a new quality of information regarding the metabolism and adaptation to the absence of key signal mechanisms in *B. subtilis* was provided. Investigations of cells lacking Pyk uncovered alterations in the import of glucose and pyruvate from the nutritional media. The concomitant consumption of both metabolites in Δpyk cells was observed, whereas in wt, the uptake of pyruvate was perceived after the complete consumption of glucose. This distinct behavior is in accordance with the pyruvate transport mechanism recently discovered in *B. subtilis*. While in wt, the pyruvate utilization suffers CCR by the CcpA-dependent glucose repressor, in Δpyk, this effect is possibly relieved due to the induction of the pyruvate facilitated transporter genes, by LytST. The lack of Pyk and consequent low intracellular pyruvate level could be involved in this induction, since LytST works in a pyruvate dose- and gradient-manner. Although the metabolic results provided help to elucidate the regulatory transport of glucose and pyruvate, complete knowledge of the pyruvate transport mechanism and the adaptation to new gradient concentrations by *B. subtilis* is yet to be determined. Other prominent pathways were affected by the *pyk* mutation such as the overflow metabolism, TCA cycle, and the PPP. Considering that pyruvate is a link of essential pathways and its fate is important for cell robustness and viability, the metabolic approach of this study helps to unravel the control of pyruvate homeostasis and *B. subtilis* adaptation to environmental challenges.

## Figures and Tables

**Table 1:** Glucose and pyruvate concentrations (mM) present in the supernatant during the time cultivation of wt and Δpyk. Data are shown as the mean values ± SD of quadruplicate samples.

Time (min)	wt				Δpyk			
	Glucose		Pyruvate		Glucose		Pyruvate	
	Concentration (mM)	SD	Concentration (mM)	SD	Concentration (mM)	SD	Concentration (mM)	SD
0	8.59	0.65	28.25	1.85	8.93	0.53	27.80	1.52
60	8.62	0.17	28.10	0.98	9.09	0.20	28.16	0.48
120	7.98	0.38	28.80	0.49	8.88	0.33	28.10	0.93
180	7.05	1.04	28.93	0.62	8.63	0.31	28.41	0.99
240	5.31	0.73	28.91	0.98	8.20	0.48	28.33	0.44
300	2.82	1.09	28.49	0.38	7.49	0.41	27.55	0.82
360	0.00	0.68	27.24	0.81	6.41	0.80	25.94	0.73
420	0.00	0.00	22.34	2.35	4.90	1.33	24.69	1.55
480	0.00	0.04	15.32	2.24	3.35	1.91	22.53	2.35
540	0.01	0.01	8.14	2.10	0.20	1.20	16.03	4.51
600	0.00	0.02	1.59	1.39	0.05	0.04	11.14	4.20
660	0.02	0.02	0.20	0.04	0.03	0.03	2.99	2.25

## Supplementary Materials

The following are available online at https://www.mdpi.com/2218-1989/9/10/216/s1, Table S1: OD of wt and Δpyk at 600 nm during growth in M9GlcPyr at 37 °C and 300 rpm, Table S2: Physiological parameters (growth rate, acetate, acetoin, and 2,3-butanediol maximum concentration) of wt and Δpyk of four replicates, Table S3: Intracellular metabolome data of *B. subtilis* wt and Δpyk. Mean of the relative amount of intracellular metabolites, SD values, and FC of four biological replicates of wt and Δpyk, Unpaired *t*-tests were also determined for each metabolite, where *p*-value represents the statistical result, Figure S1: Experimental workflow for metabolome analysis, Table S4: MS source parameters used during amino acids measurements.
