# Supplementary material for: The Absence of Pyruvate Kinase Affects Glucose-Dependent Carbon Catabolite Repression in Bacillus subtilis
Source: Metabolites. 2019 Oct 4;9(10):216. doi: 10.3390/metabo9100216 (PMC6835821; doi:10.3390/metabo9100216)
Supplement: Supplementary file 1 [file metabolites-09-00216-s001.zip › metabolites-591467-supple-revise/Supplementary Material_Metabolites.docx]

| **Time (min)** | **wt** | | | | | **Δpyk** | | | |
| --- | --- | --- | --- | --- | --- | --- | --- | --- | --- |
|  | **Rep 1** | **Rep 2** | **Rep 3** | **Rep 4** | **Rep 1** | | **Rep 2** | **Rep 3** | **Rep 4** |
| 0 | 0.143 | 0.098 | 0.065 | 0.065 | 0.119 | | 0.087 | 0.058 | 0.111 |
| 60 | 0.211 | 0.155 | 0.101 | 0.100 | 0.160 | | 0.116 | 0.081 | 0.149 |
| 120 | 0.350 | 0.250 | 0.162 | 0.167 | 0.220 | | 0.159 | 0.114 | 0.210 |
| 180 | 0.510 | 0.450 | 0.279 | 0.281 | 0.300 | | 0.230 | 0.163 | 0.310 |
| 240 | 0.800 | 0.720 | 0.500 | 0.500 | 0.420 | | 0.320 | 0.233 | 0.490 |
| 300 | 1.250 | 1.240 | 0.920 | 0.930 | 0.590 | | 0.460 | 0.350 | 0.760 |
| 360 | 1.660 | 1.670 | 1.510 | 1.670 | 0.840 | | 0.710 | 0.520 | 1.100 |
| 420 | 1.510 | 1.630 | 1.500 | 1.600 | 1.210 | | 1.050 | 0.750 | 1.620 |
| 480 | 2.010 | 2.320 | 1.890 | 1.940 | 1.800 | | 1.550 | 1.140 | 2.320 |
| 540 | 2.610 | 2.890 | 2.540 | 2.670 | 2.200 | | 2.280 | 1.640 | 2.220 |
| 600 | 2.950 | 2.850 | 2.600 | 2.250 | 2.300 | | 2.220 | 2.180 | 2.770 |
| 660 | 1.740 | 1.560 | 1.400 | 2.590 | 2.800 | | 2.670 | 2.000 | 2.580 |
| 720 | 1.500 | 1.500 | 1.450 | 1.650 | 2.580 | | 2.540 | 2.200 | 2.340 |

**Supplemental Material**

**Table S1:** OD of *B. subtilis* wt and Δpyk during growth in different media. OD of wt and Δpyk at 600 nm during growth in M9GlcPyr at 37⁰C and 300 rpm.

**Table S2**: Physiological parameters of wt and Δpyk grown in M9GlcPyr. Growth rate calculated in the beginning of exponential phase. The concentrations of acetate, acetoin, and 2,3-butanediol correspond to the maximum concentration achieved for each strain during the cultivation time course. Displayed are the mean values ± SD of 4 biological replicates.

|  | **Growth rate (h^-1^)** | **Acetate max. (mM)** | **Acetoin max. (mM)** | **2,3-butanediol max. (mM)** |
| --- | --- | --- | --- | --- |
| **wt** | 0.54 ± 0.07 | 21.0 ± 1.01 | 0.59 ± 0.16 | 0.13 ± 0.04 |
| **Δpyk** | 0.37 ± 0.05 | 21.9 ± 1.48 | 1.93 ± 0.66 | 0.23 ± 0.05 |

**Table S3**: Intracellular metabolome data of *B. subtilis* wt and Δpyk. Mean of the relative amount of intracellular metabolites, SD values, and FC of four biological replicates of wt and Δpyk cultivated in M9GlcPyr. Unpaired t-tests were also determined for each metabolite, where p-value represents the statistical result.

**See Table S3.pdf**


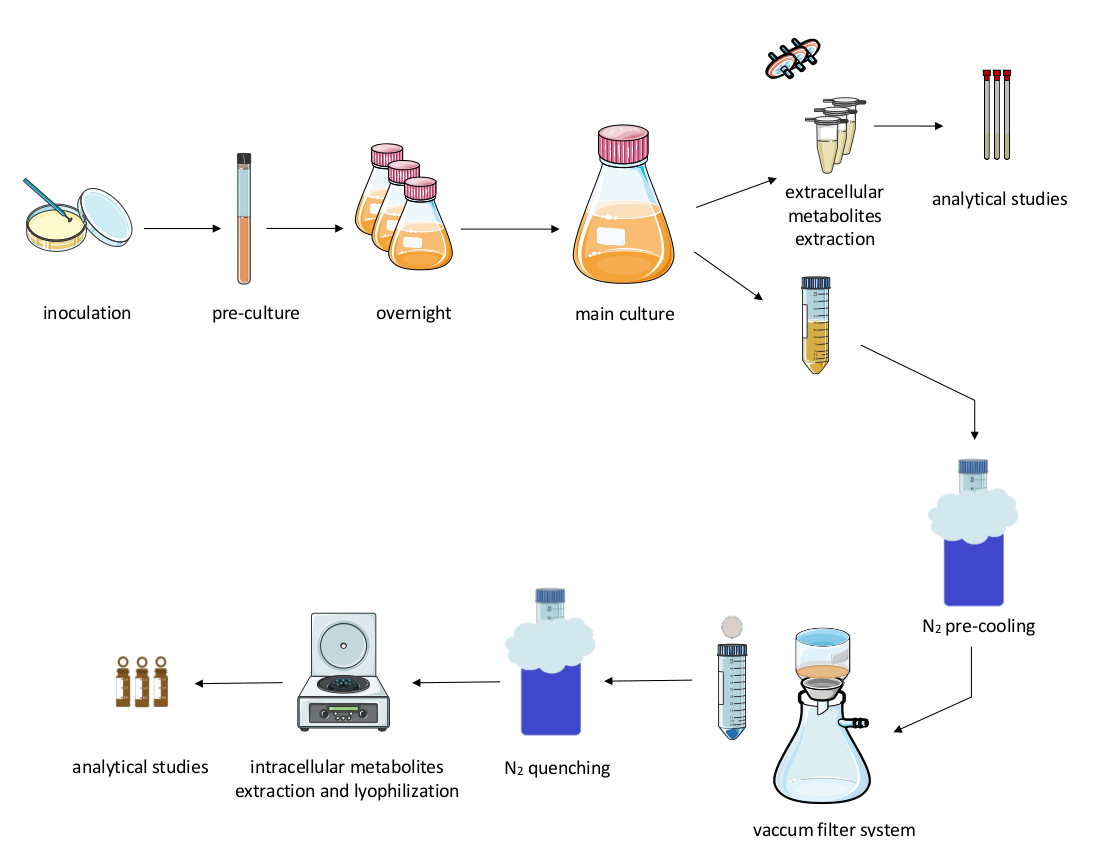


**Figure S1**: Experimental workflow for metabolome analysis. Workflow for cultivation, extracellular metabolome sampling, and intracellular sampling including cell disruption and metabolites extraction of B. subtilis cells.

**Table S4**: MS source parameters used during amino acids measurements

| **Parameters** | **Values** |
| --- | --- |
| Gas flow | 10 ml.min^-1^ |
| Gas temperature | 350 °C |
| Nebulizer pressure | 40 psi |
| Sheath gas flow | 12 l.min^-1^ |
| Sheath gas temperature | 350 °C |
| Capillary voltage | 2000 V |
| Nozzle voltage | 0 V |
