# Supplementary material for: The Absence of Pyruvate Kinase Affects Glucose-Dependent Carbon Catabolite Repression in Bacillus subtilis
Source: Metabolites. 2019 Oct 4;9(10):216. doi: 10.3390/metabo9100216 (PMC6835821; doi:10.3390/metabo9100216)
Supplement: Supplementary file 1 [file metabolites-09-00216-s001.zip › metabolites-591467-supple-revise/Table S3.pdf]

Metabolite	M9GlcPyr						
	**wt**		**Δpyk**				
	Mean	SD	Mean	SD	FC (Δpyk/wt)	log_2FC	p-value
2-oxoglutarate	0.068	0.017	0.069	0.040	1.024	0.035	0.942
2-phosphoglycerate	0.035	0.020	0.111	0.062	3.185	1.671	0.057
3-hydroxybutyrate	0.005	0.002	0.007	0.002	1.275	0.350	0.303
3-phosphoglycerate	0.986	0.241	3.028	1.631	3.072	1.619	0.048
4-hydroxy-L-proline	0.006	0.005	0.017	0.031	2.984	1.577	0.502
5-methyluridine	0.006	0.002	0.003	0.002	0.466	-1.103	0.058
5-oxo-proline	9.124	3.452	5.880	2.945	0.644	-0.634	0.203
Acetyl adenylate	0.143	0.048	0.301	0.148	2.108	1.076	0.089
Acetyl-CoA	0.017	0.015	0.024	0.020	1.470	0.556	0.552
Adenylsuccinate	0.008	0.004	0.014	0.009	1.832	0.873	0.261
ADP	2.379	0.390	2.387	0.850	1.004	0.005	0.986
Alanine	1.393	0.359	0.982	0.569	0.705	-0.505	0.267
AMP	0.356	0.065	0.763	0.175	2.144	1.100	0.005
Arginine	0.548	0.997	0.051	0.010	0.093	-3.422	0.357
Asparagine	0.041	0.034	0.048	0.046	1.161	0.216	0.826
Aspartate	2.527	1.213	2.250	1.298	0.890	-0.168	0.765
ATP	5.630	0.965	3.252	1.347	0.578	-0.792	0.028
CDP	0.066	0.011	0.079	0.034	1.196	0.258	0.493
CDP-glycerol	0.258	0.080	0.196	0.106	0.760	-0.396	0.387
Citrate	1.710	0.143	1.621	0.278	0.948	-0.077	0.589
Citrulline	0.641	0.392	0.940	0.472	1.466	0.552	0.367
CMP	0.005	0.002	0.006	0.002	1.242	0.313	0.434
CTP	0.128	0.043	0.048	0.022	0.375	-1.415	0.016
Cysteine	0.001	0.001	0.002	0.001	1.784	0.835	0.240
Cytidine	0.002	0.001	0.002	0.002	1.002	0.002	0.998
dAMP	0.007	0.004	0.008	0.001	1.066	0.092	0.826
dATP	0.069	0.024	0.035	0.022	0.509	-0.974	0.083
dCDP	0.019	0.002	0.013	0.005	0.713	-0.488	0.095
dCTP	0.095	0.029	0.026	0.013	0.268	-1.900	0.004
Deoxycytidine	0.006	0.004	0.007	0.006	1.209	0.273	0.726
Dihydroxyacetone phosphate	0.025	0.010	0.057	0.028	2.299	1.201	0.075
dTDP	0.058	0.006	0.043	0.016	0.750	-0.416	0.145
dTMP	0.023	0.006	0.011	0.004	0.469	-1.094	0.015
dUMP	0.006	0.002	0.009	0.002	1.421	0.507	0.092
Erythrose 4-phosphate	0.001	0.000	0.002	0.001	1.883	0.913	0.135
FAD	0.002	0.001	0.003	0.001	1.264	0.338	0.185
Fructose 1,6bis-phosphate	0.254	0.158	0.469	0.263	1.846	0.885	0.211
Fructose 6-phosphate	0.067	0.018	0.304	0.159	4.571	2.192	0.025
Fumarate	0.423	0.224	0.610	0.433	1.443	0.529	0.472
GDP	0.175	0.020	0.252	0.057	1.441	0.527	0.044
Gluconate 6-phosphate	0.018	0.007	0.025	0.009	1.427	0.513	0.233
Glucono 1,5lactone 6-phosphate	0.005	0.002	0.004	0.002	0.650	-0.621	0.236
Glucose 6-phosphate	0.132	0.047	0.660	0.330	5.017	2.327	0.019
Glutamate	92.072	25.467	57.827	30.004	0.628	-0.671	0.132
Glutamine	1.878	1.024	1.205	0.779	0.641	-0.641	0.335
Glycerate	0.011	0.002	0.018	0.003	1.655	0.727	0.014
Glycine	0.408	0.266	0.227	0.140	0.556	-0.846	0.273

	M9GlcPyr						
	wt		Δpyk				
Metabolite	Mean	SD	Mean	SD	FC (Δpyk/wt)	log_2FC	p-value
GMP	0.022	0.003	0.040	0.012	1.833	0.874	0.026
GTP	0.739	0.202	0.482	0.231	0.652	-0.617	0.144
Histidine	0.021	0.004	0.018	0.005	0.876	-0.192	0.427
IMP	0.218	0.032	0.086	0.033	0.395	-1.341	0.001
Isocitrate	0.007	0.006	0.005	0.005	0.799	-0.324	0.753
Isoleucine	0.324	0.204	0.134	0.070	0.412	-1.280	0.127
ITP	0.467	0.093	0.266	0.112	0.571	-0.810	0.034
Lactate	0.309	0.173	0.233	0.057	0.754	-0.407	0.436
Leucine	1.216	0.336	0.731	0.379	0.601	-0.734	0.104
Lysine	0.093	0.071	0.036	0.026	0.390	-1.357	0.186
Malate	0.016	0.002	0.045	0.057	2.721	1.444	0.361
Malonyl-CoA	0.004	0.005	0.002	0.001	0.448	-1.157	0.389
Methionine	0.091	0.031	0.054	0.026	0.591	-0.758	0.112
myo-inositole	0.029	0.024	0.014	0.018	0.496	-1.011	0.368
Ornithine	0.127	0.099	0.144	0.110	1.138	0.187	0.821
Phenylalanine	0.081	0.045	0.059	0.028	0.730	-0.455	0.447
Phenylpyruvate	0.007	0.002	0.006	0.002	0.901	-0.150	0.653
Phosphoenolpyruvate	0.054	0.010	0.544	0.350	10.035	3.327	0.031
Proline	0.052	0.053	0.035	0.021	0.679	-0.558	0.578
PRPP	0.062	0.016	0.051	0.019	0.820	-0.287	0.416
Ribose/Ribulose-phosphate	0.031	0.007	0.037	0.009	1.184	0.243	0.346
SAICAR	0.027	0.012	0.025	0.014	0.916	-0.126	0.812
Sedoheptulose 7-phosphate	0.021	0.006	0.068	0.029	3.210	1.683	0.020
Serine	0.322	0.250	0.462	0.438	1.433	0.519	0.600
Shikimate 3-phosphate	0.033	0.012	0.005	0.002	0.166	-2.593	0.004
Succinate	0.205	0.004	0.172	0.086	0.836	-0.259	0.465
Threonine	0.979	0.629	0.285	0.213	0.291	-1.781	0.082
Tryptophan	0.173	0.062	0.130	0.073	0.751	-0.412	0.406
Tyrosine	0.187	0.031	0.130	0.088	0.694	-0.527	0.266
UDP	0.280	0.024	0.337	0.137	1.202	0.266	0.445
UDP-GlucNAc	1.009	0.282	0.885	0.480	0.877	-0.189	0.672
UDP-GlucNAcenolpyruvate	0.007	0.001	0.009	0.001	1.306	0.385	0.036
UDP-Glucose	0.510	0.083	0.512	0.243	1.005	0.007	0.986
UDP-Glucuronate	0.188	0.051	0.412	0.224	2.185	1.128	0.100
UDP-MurNAc	0.063	0.007	0.086	0.032	1.373	0.457	0.207
UDP-MurNAc-ala	0.005	0.003	0.007	0.004	1.340	0.423	0.451
UDP-MurNAc-ala-glu	0.009	0.001	0.011	0.004	1.341	0.424	0.212
UDP-MurNAc-ala-glu-pm-ala-ala	0.232	0.067	0.209	0.067	0.898	-0.155	0.637
UMP	0.047	0.005	0.049	0.016	1.051	0.072	0.788
Urea	69.760	29.362	60.062	12.570	0.861	-0.216	0.566
UTP	1.668	0.572	0.668	0.317	0.400	-1.321	0.022
Valine	2.573	0.571	1.369	0.976	0.532	-0.910	0.077
XMP	0.015	0.004	0.014	0.003	0.888	-0.172	0.519
XTP	0.066	0.017	0.042	0.019	0.635	-0.655	0.107
